# Oxidatively modified forms of albumin in patients with risk factors of metabolic syndrome

**DOI:** 10.1007/s40618-014-0111-8

**Published:** 2014-06-24

**Authors:** E. Żurawska-Płaksej, E. Grzebyk, D. Marciniak, A. Szymańska-Chabowska, A. Piwowar

**Affiliations:** 1 Department of Pharmaceutical Biochemistry, Faculty of Pharmacy, Wroclaw Medical University, Borowska St. 211A, 50-556 Wroclaw, Poland; 2 Department of Drugs Form Technology, Faculty of Pharmacy, Wroclaw Medical University, Wroclaw, Poland; 3 Department and Clinic of Internal and Occupational Diseases and Hypertension, Faculty of Medicine, Wroclaw Medical University, Wroclaw, Poland

**Keywords:** Metabolic syndrome, Advanced oxidation protein products, Ischemia modified albumin, Oxidative stress, Serum albumin modifications

## Abstract

**Background:**

Metabolic syndrome (MetS) is a complex metabolic disease connected especially with lipid and carbohydrate disturbances. It is postulated that oxidative stress (OS) is linked to metabolic syndrome, constituting a novel component of its pathogenesis.

**Aim:**

We aimed to examine the plasma level of oxidatively modified proteins––advanced oxidation protein products (AOPP) and ischemia modified albumin (IMA)––as well as thiol (SH) groups and evaluate their connection with metabolic agents in relation to MetS prevalence.

**Subjects and methods:**

The levels of AOPP, IMA and SH groups were measured spectrophotometrically in 106 patients with MetS risk factors and in 32 control subjects.

**Results:**

The levels of examined parameters differed significantly between patients with MetS risk factors and the control group. AOPP significantly correlated with glucose (*r* = 0.30, *p* = 0.008), HDL-Ch (*r* = −0.34, *p* = 0.005), TG (*r* = 0.48, *p* < 0.001) and fibrinogen (*r* = 0.37, *p* < 0.001). The levels of AOPP and IMA increased progressively with the number of MetS risk factors, being the most significant for AOPP. The highest values of AOPP were associated with the presence of at least three risk factors. Only AOPP were an independent determinant for MetS occurrence in the studied population (OR = 2.72, *p* = 0.04). Mutual dependence between metabolic, oxidative stress and inflammatory parameters was revealed.

**Conclusions:**

Oxidative modifications of proteins are increased in MetS and accumulation of MetS risk factors enhances manifestation of OS. AOPP is the most appropriate parameter for determination of OS, with potential diagnostic value in MetS patients.

## Introduction

Metabolic syndrome (MetS), currently one of the major public health issues, is defined as a cluster of different risk factors that occur together and significantly increase the risk of coronary artery disease, stroke, atherosclerosis and type 2 diabetes. The mechanism of metabolic disturbances associated with MetS is complex, and not yet fully understood, but involves obesity, abnormal lipid and glucose levels and elevated blood pressure. Moreover, subclinical inflammation and hemostatic disturbances are also reported to be favorable conditions for MetS development [1–3]. All these abnormalities are connected in different aspects with oxidative/antioxidative imbalance. Recently, it has been claimed that oxidative stress (OS) is closely linked to MetS. Moreover, it is known that OS plays a crucial role in the injury response to hypoxia–ischemia conditions, which is manifested by excessive reactive oxygen species (ROS) generation and subsequent intensification of OS [4, 5]. Oxidative stress and exacerbation of disturbances connected with MetS constitute a novel important component of the pathogenesis of metabolic syndrome [6, 7].

ROS are able to react with most macromolecules, but proteins are affected to the highest degree. Albumin, because of its abundance in the blood, accounts for almost all of the excess plasma protein oxidation. The oxidative modification of proteins plays an important role in pathogenesis and development of various metabolic disturbances [8, 9]. A growing body of evidence supports the concept that protein modifications are connected with the increase of MetS prevalence. While the role of protein oxidation is well established in obesity, diabetes mellitus, arterial hypertension and dyslipidemia, only a few data concern protein oxidation in MetS [10, 11]. Therefore, we decided to estimate the plasma levels of two modified forms of albumin––advanced oxidation protein products (AOPP) and ischemia modified albumin (IMA)––which are considered as representative markers of oxidative stress intensity and degree of oxidative damage of proteins in numerous diseases [12, 13].

Advanced oxidation protein products (AOPP) are an oxidatively modified form of proteins (mainly albumin) created as the result of excessive generation of ROS and reactive chlorine species (mainly chloramine produced by myeloperoxidase in activated neutrophils). These highly reactive agents cause structural and functional changes of the albumin molecule, leading to the reduction of its antioxidant properties, and predisposing to its aggregation and deposition in tissues. AOPP are abundant in dityrosine residues, disulfide bridges, carbonyl groups and cross-links [14]. Moreover, AOPP may also cause subsequent activation of a cascade of ROS production and thereby enhance its adverse oxidizing action. The increased formation of AOPP is well documented in different diseases (e.g. diabetes, obesity, atherosclerosis) [9, 15, 16].

Ischemia modified albumin (IMA) is a modified form of albumin, created as a result of ROS action under reduced oxygen tension conditions in response to hypoxia or acidosis. During ischemia, the increased ROS generation inducing structural modification of albumin results in a reduction of its ability to bind endogenous and exogenous ions. Modification of its N-terminal amino acids (Asp-Ala-His) leads to decreased transition metal (Co^2+^, Ni^2+^, Cu^2+^) binding capacity of the albumin molecule, which is the basis of its measurement in biological fluids [17]. The most common condition connected with such disturbances is tissue ischemia caused mainly by myocardial infarction and ischemic heart disease. Moreover, increased levels of IMA are observed in diabetes (especially when complicated with hypertension), atherosclerosis and intense physical exertion [18–20].

Another effect of ROS action is oxidation of sulfhydryl groups (SH) in albumin and their depletion in blood, which subsequently exacerbates functional and structural disturbances of attacked macromolecules [21].

To our knowledge, in the currently available literature, there is no information on the plasma levels of above-mentioned oxidatively modified forms of albumin, that is AOPP and IMA, in terms of their connection with risk factors of metabolic syndrome [5, 22]. It is still unclear whether the accumulation of factors related to MetS increases the OS. Following these assumptions, in this study, we estimated plasma levels of advanced oxidation protein products and ischemia modified albumin as well as SH groups in patients with different numbers of MetS risk factors and in a control group. We also examined the connection of these modified forms of albumin with different metabolic agents with regard to occurrence of MetS.

## Materials and methods

### Patients

In this study, 106 patients (58 female and 48 male) of the Department and Clinic of Internal and Occupational Diseases and Hypertension of Wroclaw Medical University, with different numbers of risk factors of metabolic syndrome, were involved. Exclusion criteria for the study included: current smokers, acute inflammation state, current cardiovascular events, malignant and liver diseases. Thirty-two healthy controls (17 female and 15 male) were recruited from the staff of Wroclaw Medical University on the occasion of periodic health examinations. Routine medical check-ups showed no evidence of inflammation or abnormalities in lipid and carbohydrate metabolism, hypertension or kidney disorders. The anthropometric and biochemical characteristics of all participants are given in Table 1. The study was approved by Wroclaw Medical University Bioethics Committee. All participants were informed about the aim of these investigations and they gave consent to participate in this study.

**Table 1:** Anthropometric and biochemical characteristics of all patients (with different numbers of metabolic syndrome risk factors) and control subjects

Parameters	All patients (*n* = 106)	Control subjects (*n* = 32)	Differences between groups
Age (years)	55.48 ± 15.92	53.08 ± 13.09	*p* = 0.3466
BMI (kg/m^2^)	29.41 ± 5.44	25.31 ± 2.81	***p*** **=** **0.0096**
WC (cm)	89.72 ± 10.93	78.34 ± 7.44	***p*** **<** **0.001**
SBP (mmHg)	148.74 ± 31.81	122.05 ± 11.39	***p*** **<** **0.001**
DBP (mmHg)	88.49 ± 17.63	75.05 ± 7.68	***p*** **<** **0.001**
ESR (mm)	15.51 ± 11.36	9.62 ± 5.98	*p* = 0.0656
CRP (mg/L)	3.57 ± 2.08	2.25 ± 1.72	***p*** **<** **0.001**
Fibrinogen (g/L)	3.97 ± 1.91	3.45 ± 0.11	*p* = 0.2605
WBC (10^9^/L)	7.14 ± 2.10	6.65 ± 1.63	*p* = 0.2910
Platelet (10^9^/L)	229.24 ± 71.94	238.58 ± 76.54	*p* = 0.8574
Glucose (mmol/L)	5.93 ± 1.70	5.16 ± 0.38	***p*** **=** **0.0201**
Total-Ch (mmol/L)	5.34 ± 1.54	4.76 ± 0.67	*p* = 0.0812
HDL-Ch (mmol/L)	1.27 ± 0.36	1.55 ± 0.34	***p*** **<** **0.001**
LDL-Ch (mmol/L)	3.09 ± 1.18	2.79 ± 0.64	*p* = 0.1357
TG (mmol/L)	1.56 ± 0.77	1.19 ± 0.29	***p*** **=** **0.0332**
Creatinine (μmol/L)	94.68 ± 39.45	93.36 ± 12.67	*p* = 0.1074

*BMI* body mass index, *CRP* C-reactive protein, *DBP* diastolic blood pressure, *ESR* erythrocyte sedimentation rate, *HDL-Ch* HDL cholesterol, *LDL-Ch* LDL cholesterol, *SBP* systolic blood pressure, *TG* triglyceride, *Total-Ch* total cholesterol, *WBC* white blood cells, *WC* waist circumference

A two-tailed *p* value of less than 0.05 was considered significant (bolded)

According to the International Diabetes Federation recommendation [23], the diagnostic criteria for metabolic syndrome were applied. MetS was diagnosed when at least three of the following five factors were present: abdominal obesity described by waist circumference (WC ≥80 cm for women and ≥94 cm for men), high-density lipoprotein cholesterol (HDL-Ch) concentration below 50 mg/dL (1.3 mmol/L) for women and 40 mg/dL (1.0 mmol/L) for men, triglyceride (TG) concentration ≥150 mg/dL (1.7 mmol/L) or current treatment for triglyceride abnormalities, blood pressure (BP) ≥130/85 mmHg (or current use of antihypertensive medications) and fasting glucose ≥100 mg/dL (5.6 mmol/L) or previously diagnosed type 2 diabetes. Among the studied population, 47 % of patients received hypotensive agents, 24 % hypoglycemic and 29 % antidyslipidemic ones. Diabetes type 2 was previously recognized in 31 patients and atherosclerosis in 34.

On the basis of MetS diagnostic criteria, patients were divided into three groups according to the number of risk factors of metabolic syndrome: group A––with 1 or 2 risk factors of metabolic syndrome; group B––with 3 MetS risk factors; and group C––with 4 or 5 risk factors of MetS. Plasma levels of modified forms of albumin, that is AOPP and IMA, as well as SH groups, were analyzed in these groups. Furthermore, patients were divided into subgroups according to quartiles of increasing concentration of AOPP and also according to the presence of diabetes or atherosclerosis (details in “Results” section).

### Laboratory measurements

The laboratory parameters (given in Table 1) were immediately measured in blood samples collected by venous puncture technique after an overnight fast (8–12 h) in appropriate tubes (Sarstedt AG&Co, Germany) with or without an anticoagulant agent, and specimens were routinely centrifuged at 2,500×*g* for 10 min to obtain plasma or serum. In the whole blood, the number of white blood cells (WBC), platelets and erythrocyte sedimentation rate (ESR) were estimated. The serum was used to assess total cholesterol (Total-Ch), HDL-Ch and low-density lipoprotein cholesterol (LDL-Ch), triglycerides, creatinine and C-Reactive Protein (CRP). The glucose and fibrinogen concentrations were measured in plasma samples (collected into tubes with EDTA and sodium fluoride and citrate, respectively). These routine biochemical parameters were measured by standard methods using Cobas Mira Plus (Roche Diagnostics, Basel, Switzerland) and Konelab 20i (Thermo Scientific, Waltham, USA) automated analyzers.

Levels of modified forms of proteins (AOPP and IMA) as well as SH groups and albumin concentrations were measured in plasma samples (collected into tubes containing 16 IU/mL heparin). These plasma samples were immediately frozen and stored at −85 °C (not longer than 3 months) until the simultaneous determinations of these parameters. All determinations were done in duplicate. AOPP were measured spectrophotometrically according to the method by Witko-Sarsat et al. [24] described previously [15]. IMA was also measured spectrophotometrically according to Bar-Or et al. [25] as described previously [19]. The concentration of SH groups was determined using 5-5′-dithio-bis(2-nitrobenzoic acid) solution (DTNB) according to the method of Rice-Evans et al. [26], as described previously [15]. The bromocresol purple (BCP) dye-binding method was used for the estimation of serum albumin [27].

### Statistical analysis

Data are expressed as mean ± standard deviation (X ± SD). The statistical analysis was performed using Statistica PL for Windows (v.10.0). The normality of distribution of all variables was evaluated by Shapiro–Wilk test. Comparisons between examined groups were performed by Mann–Whitney *U* test or Student’s *t* test. Differences between groups of patients with different numbers of risk factors of MetS were evaluated by analysis of variance (ANOVA) followed by multiple comparison post hoc Fisher test. Spearman rank correlations were used to test the relationships of AOPP, IMA and SH with routine laboratory parameters. To identify independent factors for the presence of MetS, multivariate analysis was performed and the effect size was reflected as the odds ratio (OR) with 95 % confidence interval (CI). Correspondence analysis was performed to evaluate the association between number of MetS risk factors and quartiles of AOPP. Canonical analysis was used to evaluate the influence of modified forms of albumin (AOPP and IMA) together with inflammatory parameters on MetS risk factors. A value of *p* less than 0.05 was considered as statistically significant.

## Results

The parameters of anthropometric and biochemical characteristics of the study participants (given in Table 1), as expected, varied between patients with MetS risk factors and the control group. The BMI and waist circumference, systolic and diastolic blood pressure, CRP, fasting glucose as well as triglycerides were significantly higher and HDL cholesterol was lower in these patients. However, age, WBC and platelet count, ESR, fibrinogen, creatinine as well as total cholesterol and LDL-Ch did not show significant differences. From Spearman correlation analysis, it was revealed that AOPP were associated with glucose (*r* = 0.30, *p* = 0.0076), HDL-Ch (*r* = −0.34, *p* = 0.0046), TG (*r* = 0.48, *p* < 0.001), and fibrinogen (*r* = 0.37, *p* = 0.0004). IMA was not associated with any routine biochemical parameter and SH groups correlated only with fibrinogen (*r* = −0.34, *p* = 0.0054).

Data concerning modified forms of albumin (AOPP and IMA) as well as SH groups in control subjects (without MetS risk factors) and in patients with MetS risk factors, also subdivided into three groups, according to the number of MetS risk factors (A––with 1 or 2 risk factors, B––with 3 risk factors, C––with 4 or 5 risk factors of MetS) are presented in Table 2. Significant differences between control subjects and each group of patients are observed in all analyzed parameters. Almost 60 % increase in AOPP concentration, 70 % in IMA level and about 20 % decrease in SH concentration (*p* < 0.001, 0.001 and 0.05, respectively) were observed in MetS patients, while concentration of plasma albumin did not differ. Analyzing differences between groups of patients with different numbers of MetS risk factors (A, B and C), we also found no differences in albumin concentration. However, concentration of AOPP increased progressively with the increasing number of risk factors and significant differences were observed between group C and each of the remaining groups. The level of IMA was somewhat higher in groups B and C than in group A, but the increase was not significant. Concentration of SH groups had a trend to decrease slightly but without statistical significance.

**Table 2:** The levels of albumin and its modified forms (AOPP and IMA) and SH groups in plasma of control subjects and patients (all and in groups A–C divided on the basis of MetS risk factors number)

Parameters	Control subjects (*n* = 32)	All patients (*n* = 106)	Group A (1–2 risk factors) (*n* = 39)	Group B (3 risk factors) (*n* = 31)	Group C (4–5 risk factors) (*n* = 36)	Statistical significance (ANOVA)
Albumin (g/L)	43.18 ± 7.09	41.32 ± 6.29	43.24 ± 5.64	41.68 ± 6.54	40.04 ± 8.62	*p* = 0.876
AOPP (μmol/L)	90.44 ± 30.39	142.44 ± 69.00	117.44 ± 42.21	129.69 ± 41.71	180.52 ± 80.02	*p* < 0.001
		*p* < 0.001^C^	*p* = 0.049^C^	*p* = 0.013^C^	*p* < 0.001^C^	
					*p* = 0.002^A^	
					*p* = 0.014^B^	
IMA (ABSU)	0.315 ± 0.093	0.530 ± 0.153	0.494 ± 0.133	0.553 ± 0.152	0.552 ± 0.144	*p* < 0.001
		*p* < 0.001^C^	*p* < 0.001^C^	*p* < 0.001^C^	*p* < 0.001^C^	
SH groups (μmol/L)	664.90 ± 112.56	552.39 ± 140.58	556.48 ± 123.48	552.98 ± 139.39	547.48 ± 149.97	*p* = 0.025
		*p* = 0.015^C^	*p* = 0.014^C^	*p* = 0.015^C^	*p* = 0.023^C^	

Significant differences versus: ^C^ control group, ^A^ patients from group A, ^B^ patients from group B (assessed by Mann–Whitney *U* test or Fischer post hoc test)

A two-tailed *p* value of less than 0.05 was considered significant

**Fig. 1:**
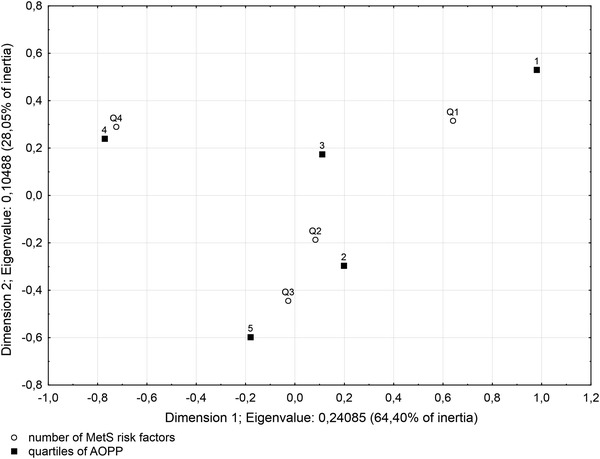
Association between quartiles of AOPP concentration and number of MetS risk factors in 2-dimensional model

On the basis of the above observations, we carefully examined the association of AOPP concentration with number of MetS risk factors. Quartiles of AOPP concentration (Q1–Q4) were calculated and patients were divided into four subgroups according to increasing values of AOPP: quartile 1 (Q1) included values of AOPP ≤99.29 μmol/L, quartile 2 (Q2) >99.29 and ≤124.65 μmol/L, quartile 3 (Q3) >124.65 and ≤158.28 μmol/L, and quartile 4 (Q4) >158.28 μmol/L. The variable describing the number of MetS risk factors was categorized into five subgroups (from 1 to 5 risk factors). In correspondence analysis, we constructed a 2-dimensional model (*χ* ^2^ = 28.421, d*f* = 12, *p* = 0.005), which explained 92.45 % of the total inertia (the first dimension explained 64.40 % and the second 28.05 % of total inertia). An illustration of this analysis is presented in Fig. 1. Interpreting this plot, groups “4” and “1” (for number of MetS risk factors) and “Q4” and “O1” (for quartiles of AOPP) had the greatest participation of total inertia in the first dimension. Groups “5” and “1” (for number of MetS risk factors) and “Q3” and “O1” (for quartiles of AOPP) had the greatest participation of total inertia in the second dimension. Statistical analysis of the final configuration of points demonstrated that 71 % of patients with only one MetS risk factor belong to Q1 of AOPP concentration and 58 % of patients with four MetS risk factors belong to Q4. In turn, 52 % of patients from Q4 had four MetS risk factors, and 26 % had three of them. Furthermore, among patients from Q1, only 5 % had more than three MetS risk factors and among patients with one MetS risk factor there is nobody from Q3 and Q4.

**Fig. 2:**
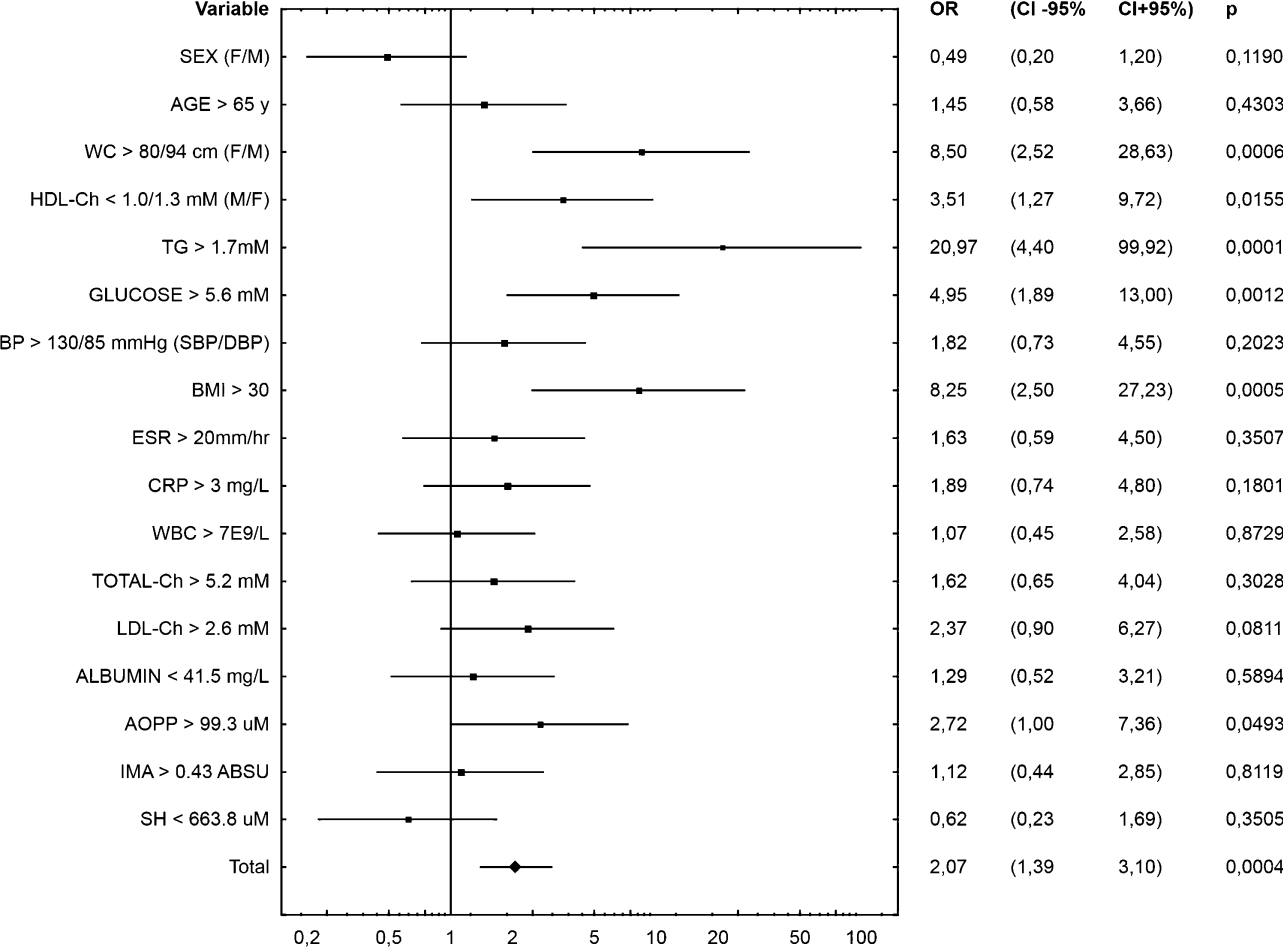
Multivariate analysis for independent determinants of metabolic syndrome

Multivariate analysis for independent determinants of metabolic syndrome presence is presented in Fig. 2. We compiled conventional MetS risk factors with parameters of albumin modification examined by us. The following variables were categorized: sex, age, WC, HDL-Ch, TG, glucose, BP, as well as AOPP, IMA and SH groups. As revealed, the constructed model was statistically significant (OR = 2.25, *p* = 0.0134) and variables with the greatest impact on statistical significance of this model were: hypertriglyceridemia (OR = 21, *p* < 0.001), abdominal obesity (assessed by waist circumference, OR = 8.5, *p* < 0.001), hyperglycemia (OR = 4.95, *p* = 0.0012), HDL hypocholesterolemia (OR = 3.5, *p* = 0.0155) as well as increased AOPP concentration (OR = 2.72, *p* = 0.0493). Obviously, among examined modified forms of albumin, only AOPP were an independent risk factor for metabolic syndrome occurrence in the studied population.

We also investigated whether the presence of diabetes or atherosclerosis had any influence on values of this parameter. We divided patients into subgroups according to two criteria: presence or absence of diabetes, and presence or absence of atherosclerosis. One-way analysis of variance showed that there were no significant differences between patients without MetS and with or without diabetes (108.62 vs 130.11 µmol/L) as well as between patients with MetS and with or without diabetes (155.65 vs 151.38 µmol/L). We found an increase in IMA levels in patients with MetS with concomitant atherosclerosis in comparison to patients with MetS without atherosclerosis (0.578 vs 0.533 absorbance units [ABSU]). However, in patients without MetS, the presence of atherosclerosis also had an impact on IMA level (0.537 vs 0.501 ABSU).

In addition, we correlated selected MetS risk factors, reflecting carbohydrate and lipid disorders (set of criterion variables) with selected parameters reflecting inflammation and oxidative stress (set of predictor variables) using canonical analysis. We obtained a statistically significant (*p* = 0.048) model (canonical *r* = 0.776), in which three canonical variables (CRP, AOPP and IMA) exhibited 100 % variability in this set and 100 % variability in the set of MetS risk factors (glucose, HDL-Ch and TG). AOPP had the largest canonical weight of predictor variables (0.75). Moreover, redundancy of the set of criterion variables was 34 and 43 % in the set of predictor variables.

## Discussion

It is known that MetS patients exhibit activation of biochemical pathways leading to increased production of reactive oxygen species and numerous studies describe oxidative status in individual components of MetS. In fact, OS, due to the production of ROS, impairment of antioxidant enzymatic defenses, oxidative modifications of many macromolecules (mainly proteins and low-density lipoproteins), and their deposition in tissues, organs and vascular wall, may be additional component of the pathogenesis of MetS [28–30]. There is a current theory that OS could be an early event in the pathology of the chronic diseases associated with the metabolic syndrome, not only a consequence of these disorders [7]. However, the exact mechanism of its involvement in the development of MetS remains unclear. This is partly due to the wide diversity of markers which reflect OS intensity and lack of standardization of its determination in certain cases.

In this study, we focused on selected biochemical parameters, reflecting OS-induced modifications of the albumin molecule. We chose AOPP as a marker of irreversible damage of proteins caused by OS, IMA as a marker of hypoxia-induced OS, and SH groups as a marker of redox status of albumin reflecting non-enzymatic antioxidant defense. We revealed significantly higher levels of AOPP and IMA with lower SH concentrations in patients with different numbers of metabolic syndrome features in comparison to the control group. We ascertained that plasma level of albumin had no impact on examined parameters (it was at a similar level in both examined groups of participants and there were no subjects with abnormalities in this area). Obtained results confirm that OS is increased in patients with MetS risk factors and causes direct oxidative damage to proteins in these patients. Our findings are consistent with other investigations regarding protein oxidation in patients with full-blown metabolic syndrome [11]. Caimi et al. [31] observed higher concentration of carbonyl groups in these patients and Korkmaz et al. [22] found increased AOPP levels and pro-oxidant/antioxidant balance (PAB) values. Higher IMA levels in MetS patients were observed by Valle-Gottlieb et al. [5] in association with cardiometabolic risk factors in MetS. However, to our knowledge, there is no information about plasma levels of AOPP in combination with IMA and SH groups in terms of their connection with risk factors of occurrence of MetS. Only a single investigation by Demir et al. [32] has determined simultaneously AOPP and IMA levels, but in patients with cardiac syndrome X. The authors found an increase in OS (expressed as increased values of PAB, AOPP and IMA) and a positive correlation between AOPP and IMA in these patients. In contrast, we did not detect a significant relationship between these parameters, which may be caused by different mechanisms of formation of these products. AOPP arise as a result of direct action of ROS (oxidation of amino acid residues) and subsequent structural rearrangement (creation of disulfide bridges and other cross-linking reactions). In turn, IMA is produced under hypoxic conditions. It is suggested that a pro-atherogenic environment, which develops in patients with MetS risk factors, leads to a decrease in tissue oxygen perfusion and triggers albumin modifications.

Our study showed that among modified forms of albumin, AOPP exhibited the most connections with other metabolic parameters (significant correlations with glucose, HDL-Ch, TG and fibrinogen). Korkmaz et al. [22] also found that the levels of AOPP were significantly higher (by nearly 60 %) in MetS patients than in controls and were positively correlated with glucose, HbA1c, TG and insulin levels and HOMA-IR values. In this light, the AOPP concentration is the best parameter accurately reflecting OS in patients with MetS risk factors.

As mentioned above, the role of OS in MetS is under intensive investigation; nevertheless, there are also still insufficient data to determine whether the accumulation of factors related to MetS increases the degree of underlying OS. It is only recently that Yubero-Serrano et al. [33] in a comparative cross-sectional study from the LIPGENE cohort examined the relationship between the number of MetS components and the degree of OS in MetS patients divided into four groups (based on the number of MetS components). The authors found significant differences in soluble vascular cell adhesion molecule 1, H_2_O_2_, lipid peroxidation products, ischemic reactive hyperemia, total nitrite levels and superoxide dismutase and glutathione peroxidase activities in plasma of examined patients. In the present study, we analyzed the AOPP, IMA and SH group levels in groups of patients (A–C) divided on the basis of number of recognized MetS components, and we found that concentrations of these parameters were significantly different in all groups of patients versus control subjects. Accordingly, our study showed a general tendency to elevation of protein oxidation (evidence for intensification of OS) in patients with more MetS components. It should be highlighted that statistically significant differences were observed even in patients with only one or two MetS risk factors (without recognized MetS) in comparison to the control group, which indicates that oxidative modifications of albumin are one of the first biochemical disturbances arising in response to metabolic abnormalities foretelling development of MetS. This supports the hypothesis that OS is engaged in the pathomechanism of this disease [6, 22]. Youn et al. [34] observed that mice with increased vascular ROS production (overexpression of p22phox) developed exaggerated obesity and increased fat mass, which was associated with development of glucose intolerance, reduced HDL-Ch, increased level of leptin and monocyte chemotactic protein 1. While the high prevalence of MetS underscores the need for early identification of risk and introduction of prevention efforts, determination of OS markers seems to be clinically relevant. Analyzing results obtained in particular groups, among modified forms of albumin, the largest percentage of changes was observed for IMA when comparing the control group to patients (regardless of the number of MetS risk factors). However, only AOPP concentration differed significantly between patients from group C and patients from groups A and B. We suggest that AOPP adequately reflects the cumulative effect of MetS risk factors on escalation of oxidative damage of proteins. These results were confirmed by correspondence analysis which revealed that in quartiles of AOPP (Q1–Q4), lower AOPP concentration was closely connected with the presence of fewer MetS risk factors and inversely, the greater the number of risk factors the higher the AOPP concentration. Moreover, patients belonging to Q4 of AOPP concentration usually had more than three risk factors.

Performed multivariate analysis also ascertained that AOPP levels are the most relevant OS biomarker in patients suffering from MetS and the most important independent (after adjustment for sex and age) determinant among examined modified forms of albumin. This indicates that its determination may be equally important as other conventional MetS risk factors. Sebekova et al. [10] also suggest that AOPP concentration may have predictive value to determine the degree of OS in patients with MetS risk factors. Hopps et al. [6] even suggested that AOPP measurement could be a diagnostic tool to identify patients with increased risk of MetS development However, Korkmaz et al. [22] examined the diagnostic accuracy of AOPP by receiver operating characteristic curve analysis and found it to be fairly low (61.8 % sensitivity and 60 % specificity). Nonetheless, all these findings confirm that high AOPP levels are indicative of an increase in oxidative stress and may reflect direct oxidative damage to proteins in MetS patients [35]. It has been proposed that the detrimental action of AOPP results not only from direct damage of the protein molecule leading to its functional impairment, but also from binding and activation of RAGE receptors on the surface of various cells (especially macrophages/monocytes, endothelial and vascular cells) with subsequent activation of many signaling pathways (including activation of mitogen-activated protein kinases and nuclear factor kappa B) [12, 36].

We are aware of the difficulty of interpreting these data because of heterogeneity in the studied group, especially in the context of different co-morbidities accompanying MetS. On the basis of our investigations, we know that AOPP levels are significantly higher in patients with diabetes type 2, which is also affirmed by other authors [15, 37]. Thus, an additional question that we explored in this study is whether the presence of diabetes had an impact on values of AOPP. As it was revealed, in this study, the observed increase of OS, reflected by AOPP concentration, was not influenced by diabetes. We also presumed that IMA levels may be influenced by the presence of atherosclerosis, as suggested by Kazanis et al. [38]. Atherosclerotic plaque may significantly impair blood flow in vessels, creating hypoxic conditions and favoring IMA formation. Indeed, we found higher levels of IMA in atherosclerotic patients with or without MetS. This means that IMA should be interpreted with caution and may not be a reliable marker in patients with atherosclerosis. However, it remains a differentiating factor in patients without atherosclerosis. In another study [5], it was found (by multivariate analysis) that IMA and MetS were associated independently of sex, age, diabetes mellitus 2, and hypercholesterolemia, so the conclusions are not unambiguous. In this context, IMA may represent a possible indication of progressive peripheral oxygenation insufficiency caused by vascular dysfunction in MetS patients.

Because in our study patients with MetS risk factors are characterized by higher inflammatory parameters (among which only CRP differed significantly), we linked lipid-carbohydrate MetS features with OS and inflammation in canonical analysis. We revealed that the set of variables AOPP, IMA, CRP explains 43 % of variance in the other set (glucose, HDL-Ch, TG). The observed interaction between the panel of oxidative-inflammatory parameters and metabolic ones indicates the inseparable connection between these factors and emphasizes the complexity of mutual dependences in the pathomechanism of MetS. A large body of evidence supports the concept that a state of chronic low-level inflammation may have an important role in MetS-related manifestations [39, 40]. It may be somehow connected with obesity, which is the primary MetS component. It is known that white adipose tissue secretes various inflammatory cytokines (such as TNF alpha or IL-6), which can subsequently alter insulin sensitivity by triggering different key steps in the insulin signaling pathway [41]. In aggregate, our study contributed to a better understanding of the relationships between metabolic syndrome, oxidative stress and inflammation.

We realize that this study has some limitations. The number of estimated patients was not too large and therefore we could not create representative subgroups with particular numbers of MetS risk factors. We also did not consider the impact of medications taken and dietary habits. Nevertheless, our study brings further evidence that MetS is directly linked to OS, which is reflected in increased levels of oxidatively modified forms of albumin (AOPP and IMA) and depletion of SH concentration. Moreover, it emerged clearly from our investigations that accumulation of risk factors of MetS has a significant impact on manifestation of OS. We consider AOPP concentration to be the most adequate parameter for determination of OS in patients with MetS risk factors, being correlated with important metabolic parameters and associated with the number of risk factors. Taken together, these results suggest that AOPP may play a causal role in the pathogenesis and development of MetS, and may be a promising candidate for risk assessment and a potential intervention target for MetS patients.

## List of abbreviations

DTNB: 5-5′-dithio-bis(2-nitrobenzoic acid)
AOPP: Advanced oxidation protein products
BP: Blood pressure
BCP: Bromocresol purple
CI: Confidence intervals
CRP: C-reactive protein
ESR: Erythrocyte sedimentation rate
HDL-Ch: High-density lipoprotein cholesterol
LDL-Ch: Low-density lipoprotein cholesterol
MetS: Metabolic syndrome
OR: Odds ratio
OS: Oxidative stress
PAB: Pro-oxidant/antioxidant balance
Q: Quartile
ROS: Reactive oxygen species
SD: Standard deviation
SH groups: Thiol groups
Total-Ch: Total cholesterol
TG: Triglycerides
WBC: White blood cells
